# Reston Ebolavirus Antibodies in Bats, the Philippines

**DOI:** 10.3201/eid1708.101693

**Published:** 2011-08

**Authors:** Satoshi Taniguchi, Shumpei Watanabe, Joseph S. Masangkay, Tsutomu Omatsu, Tetsuro Ikegami, Phillip Alviola, Naoya Ueda, Koichiro Iha, Hikaru Fujii, Yoshiyuki Ishii, Tetsuya Mizutani, Shuetsu Fukushi, Masayuki Saijo, Ichiro Kurane, Shigeru Kyuwa, Hiroomi Akashi, Yasuhiro Yoshikawa, Shigeru Morikawa

**Affiliations:** Author affiliations: University of Tokyo, Tokyo, Japan (S. Taniguchi, S. Watanabe, N. Ueda, K. Iha, H. Fujii, Y. Ishii, S. Kyuwa, H. Akashi, Y. Yoshikawa);; National Institute of Infectious Diseases, Tokyo (S. Taniguchi, S. Watanabe, T. Omatsu, K. Iha, T. Mizutani, S. Fukushi, M. Saijo, I. Kurane, S. Morikawa);; University of the Philippines, Laguna, the Philippines (J.S. Masangkay, P. Alviola);; University of Texas of Medical Branch, Galveston, Texas, USA (T. Ikegami)

**Keywords:** Reston Ebolavirus, antibodies, bats, filoviruses, Ebolavirus, Ebola, viruses, the Philippines, letter

**To the Editor:** Filoviruses cause highly lethal hemorrhagic fever in humans and nonhuman primates, except for Reston Ebolavirus (REBOV), which causes severe hemorrhagic fever in macaques (*1**,**2*). REBOV epizootics among cynomolgus macaques occurred in 1989, 1990, 1992, and 1996 (*2*) and among swine in 2008 (*3*). African fruit bats have been suggested to be natural reservoirs for Zaire Ebolavirus and Marburg virus (*4**–**6*). However, the natural reservoir of REBOV in the Philippines is unknown. Thus, we determined the prevalence of REBOV antibody–positive bats in the Philippines.

Permission for this study was obtained from the Department of Environment and Natural Resources, the Philippines, before collecting bat specimens. Serum specimens from 141 wild-caught bats were collected at several locations during 2008–2009. The bat species tested are summarized in the Table. Captured bats were humanely killed and various tissues were obtained. Carcasses were then provided to the Department of Environment and Natural Resources for issuance of a transport permit.

**Table:** REBOV-specific IgG in *Rousettus amplexicaudatus* bats and other bats, the Philippines*

Bat ID	Collection site	ELISA optical density			IFA titer	
		REBOV NP	REBOV GP		REBOV NP	REBOV GP
1539	FD	**2.13**	–0.21		**1,280**	<20
1632	FQ1	**0.88**	0.2		<20	<20
1642	FQ1	0.36	**5.22**		<20	**20**
1643	FQ1	**1.26**	**0.92**		<20	<20
1651	FQ1	**1.61**	**1.02**		<20	<20
1657	FQ1	–0.45	**1.69**		<20	<20
1660	FQ1	**3.8**	**2.51**		**640**	<20

*Cutoff optical density of ELISA was 0.82 (sum of optical densities at serum dilutions of 1:100, 1:400, 1:1,600, and 1:6,400). Values in **boldface** are positive results. REBOV, Reston Ebolavirus; Ig, immunoglobulin; IFA, indirect immunofluorescence assay; ID, identification; NP, nucleoprotein; GP, glycoprotein; FD, forest of Diliman at the University of the Philippines Diliman campus; FQ1, forest at the Agricultural College in Province of Quezon, the Philippines. The other 9 *R. amplexicaudatus* bats collected at FQ1 had negative results for all assays. The following bat species also had negative results: 5 *Eonycteris spelaea,* 35 *Cynopterus brachyotis,* 38 *Ptenochirus jagoli,* 6 *Haplonycteris fischeri,* 2 *Macroglossus minimus,* 2 *Rhinolophus rufus,* 1 *Rhinolophus arcuatus*, 9 *Emballonura alecto,* 2 *Pipistrellus javanicus*, 5 *Scotophilus kuhlii,* 8 *Miniopterus australis,* 8 *M. schreibersi,* 1 *M. tristis tritis,* 1 *Hipposideros diadema,* 1 *Myotis macrotarsus*, and 1 bat of unknown species.

We used immunoglobulin (Ig) G ELISAs with recombinant nucleoprotein (NP) and glycoprotein (GP) of REBOV (*7*) to determine REBOV antibody prevalence. REBOV NP and GP were expressed and purified from Tn5 cells infected with recombinant baculoviruses AcResNP and AcResGPDTM, which express NP and the ectodomain of GP with the histidine tag at its C-terminus. We also used histidine-tagged recombinant Crimean-Congo hemorrhagic fever virus NP as a negative control antigen in the IgG ELISA to confirm specificity of reactivity.

In IgG ELISAs for bat specimens, positive results were detected by using rabbit anti-bat IgG and horseradish peroxidase–conjugated anti-rabbit IgG. Anti-bat (*Rousettus aegyptiacus*) rabbit IgG strongly cross-reacts with IgGs of other bat species, including insectivorous bats (*8*). Bat serum samples were 4-fold serially diluted (1:100–1:6,400) and tested by using IgG ELISAs. Results of IgG ELISAs were the sum of optical densities at serum dilutions of 1:100, 1:400, 1:1,600, and 1:6,400. Cutoff values (0.82 for both IgG ELISAs) were determined by using serum specimens from REBOV antibody–negative bats.

Among 16 serum samples from *R. amplexicaudatus* bats, 5 (31%) captured at either the forest of Diliman (14°38′N, 121°2′E) or the forest of Quezon (14°10′N, 121°50′E) had positive results in the IgG ELISA for REBOV NP, and 5 (31%) captured at the forest of Quezon had positive results in the IgG ELISA for REBOV GP. The REBOV NP antibody–positive bats serum samples were confirmed to be NP antibody positive in the IgG ELISA by using glutathione-S-transferase–tagged partial REBOV NP antigen (*9*). Three samples had positive results in both IgG ELISAs (Table). Serum samples from other bat species had negative results in IgG ELISAs.

All bat serum samples were also tested by indirect immunofluorescence assays (IFAs) that used HeLa cells expressing NP and GP (*10*). In the IFAs, 2 samples from *R. amplexicaudatus* bats captured at the forest of Diliman and the forest of Quezon had high titers (1,280 and 640, respectively) of NP-specific antibodies, and 1 sample from an *R. amplexicaudatus* bat captured at the forest of Quezon had a positive result in the GP-specific IFA (titer 20). All IFA-positive samples were also positive in the IgG ELISA (Table).

The forest of Diliman is ≈30 km from the monkey facility and the Bulacan farm where REBOV infections in monkeys and swine, respectively, were detected. The forest of Quezon is ≈60 km from the monkey facility. Samples from other bat species had negative results in IFAs. We also performed heminested reverse transcription PCR specific for the REBOV NP gene with spleen specimens from all 16 *R. amplexicaudatus* bats but failed to detect any REBOV-specific amplicons.

REBOV-specific antibodies were detected only in *R. amplexicaudatus* bats, a common species of fruit bat, in the Philippines. In Africa, *R. aegyptiacus* bats*,* which are genetically similar to *R. amplexicaudatus* bats, have been shown to be naturally infected with Zaire Ebolavirus and Marburg virus. Thus, *R. amplexicaudatus* bats are a possible natural reservoir of REBOV. However, only 16 specimens of *R. amplexicaudatus* bats were available in this study, and it will be necessary to investigate more specimens of this species to detect the REBOV genome or antigens to conclude the bat is a natural reservoir for REBOV.

We have shown that *R. amplexicaudatus* bats are putatively infected with REBOV or closely related viruses in the Philippines. Antibody-positive bats were captured at the sites near the study areas, where REBOV infections in cynomolgus monkeys and swine have been identified. Thus, bats are a possible natural reservoir of REBOV. Further analysis to demonstrate the REBOV genome in bats is necessary to conclude that the bat is a reservoir of REBOV.
